# Integrated Control of *Aedes albopictus* in a Residential Area Through a Community-Based Approach: NESCOTIGER, a Large-Scale Field Trial in Valencia, Spain †

**DOI:** 10.3390/pathogens14040367

**Published:** 2025-04-08

**Authors:** Marcos López-de-Felipe, Pedro María Alarcón-Elbal, Isaac García-Masiá, Anna Flor-Sánchez, Pilar Mateo-Herrero, Juan Pablo Serna-Mompeán, Juan Pablo Orán-Cáceres, Rubén Bueno-Marí, Ignacio Gil-Torró

**Affiliations:** 1 R&D Department, Laboratorios Lokímica S.A., Ronda Auguste y Louis Lumière, 23, Nave 10, 46980 Paterna, Valencia, Spain; marcos.lopez@isciii.es (M.L.-d.-F.); isaac.garcia@rentokil-initial.com (I.G.-M.); jserna@lokimica.es (J.P.S.-M.); joran@lokimica.es (J.P.O.-C.); ruben.bueno@rentokil-initial.com (R.B.-M.); 2 Laboratory of Medical Entomology, National Center for Microbiology, Instituto de Salud Carlos III, Ctra. de Pozuelo, 28, 28222 Majadahonda, Madrid, Spain; 3 Research Group on Vector-Borne Zoonoses (ZOOVEC), Department of Animal Production and Health, Veterinary Public Health and Food Science and Technology (PASAPTA), School of Veterinary Medicine, Universidad Cardenal Herrera-CEU, CEU Universities, c/Santiago Ramón y Cajal, 46115 Alfara del Patriarca, Valencia, Spain; 4 European Center of Excellence for Vector Control, Rentokil Initial, Ronda Auguste y Louis Lumière, 23, Nave 10, 46980 Paterna, Valencia, Spain; 5 Independent Researcher; annaflorschz@gmail.com; 6 R&D Department, Inesfly Corporation S.L., Cno. Pascualeta, 5, 46200 Paiporta, Valencia, Spain; pilar.mateo@inesfly.com (P.M.-H.); ignacio.gil@inesfly.com (I.G.-T.); 7 Parasite & Health Research Group, Department of Pharmacy, Pharmaceutical Technology and Parasitology, University of Valencia, Av. Vicent Andrés Estellés, s/n, 46100 Burjassot, València, Spain; 8 Research Group on Vector-Borne Zoonoses (ZOOVEC), Department of Pharmacy, School of Health Sciences, Universidad Cardenal Herrera-CEU, CEU Universities, c/Santiago Ramón y Cajal, 46115 Alfara del Patriarca, Valencia, Spain

**Keywords:** NESCOTIGER, mass trapping, *Aedes albopictus*, citizen science, control, field trial, community intervention

## Abstract

*Aedes albopictus* has established populations in several European countries with a sustained spreading pattern through the continent. This invasive mosquito is a public health threat due to its vector competence for multiple arboviruses. Notably, the peri-domestic habits of this hematophagous insect greatly diminish the efficacy of regular control activities, as individuals may harbor in private areas. The oviposition behavior can be exploited for targeting adults and immature stages through different types of traps. An experimental integrated control program, which included a community-based mass trapping intervention in private areas, control of public street-catch basins, and an educational campaign, was developed in an infested residential area in Valencia (Eastern Spain). Focusing on mass trapping, participating residents deployed traps belonging to three modes of action in their gardens during the mosquito season. A total of 1028 families participated in the project, and 2884 traps were deployed. The study sector where adult lethal ovitraps were used showed the lowest adult collections, and residents living in this sector reported the highest satisfaction rates in a perception survey. The mass deployment through a community-based approach of the adulticidal oviposition trap type appears to be a promising tool for controlling *Ae. albopictus* in residential areas.

## 1. Introduction

Since the end of the last century, *Aedes albopictus* (Skuse, 1894) (Diptera: Culicidae) has steadily and rapidly spread across the world from its original habitat in Southeast Asia [1]. In Europe, it was first detected in 1979 in Albania [2], after which multiple introduction events from different mosquito populations have been suspected across the continent. By July 2024, this *Aedes* invasive mosquito had established populations in at least 14 European countries [3], including Spain, where it has quickly disseminated since its detection in 2004 [4,5,6]. Human-mediated transportation [7,8] and phenotypic and ecological plasticity of the species have enabled such a rapid colonization of new territories, mainly in urban environments [9].

In addition to its biting annoyance, *Ae. albopictus* is known to be competent for the transmission of several arboviruses [10] such as dengue, Zika, and chikungunya [11]. Not accounting for the largest autochthonous dengue outbreak in Europe, with 2168 probable cases recorded in the island of Madeira (Portugal) [12], where *Aedes aegypti* (Linnaeus, 1762) was established as the sole vector [13], a total of 941 autochthonous *Aedes*-borne arbovirus infections have been documented in the continent between 2000 and 2023 (200 DENV; 738 CHIKV; 3 ZIKV) [14]. Furthermore, autochthonous cases of dengue were detected in Spain in 2018, 2019, 2022, and 2024 [15], confirming the public health threat linked with the establishment of *Ae. albopictus* in many regions of this country.

The exophilic and exophagic traits of this mosquito [16], in addition to its cryptic breeding sites and skip-oviposition behavior [17], seriously challenge traditional control methods. In this context, interventions solely based on adulticidal spraying treatments are regarded in most cases as ineffective [18] or inadequate due to their environmentally negative impacts. The mosquitoes’ foci in residential areas make them inaccessible to public services [19], greatly jeopardizing the effectiveness of management interventions conducted by local public health personnel. As such, citizen participation in combination with local authorities and private parties is generally considered essential for the success of vector control interventions against *Ae. albopictus* [20].

Notably, though, community participation through educational campaigns did not cause an impact on the number of *Ae. albopictus* breeding sites in New Jersey (USA) [21]. Additionally, source reduction as a unique intervention appears to be insufficient despite widespread campaigns being conducted by municipalities [22].

On the other hand, vector control interventions conducted by public services mostly focus on the treatment of street catch basins (SCB) with insect growth regulators (IGRs), such as diflubenzuron [23], and/or biological larvicides, such as the entomopathogenic bacteria *Bacillus thuringiensis* var. *israelensis* (*Bti*) [24]. Both strategies target immature stages that breed on the remaining water. Within this context, public control interventions against larvae and/or adult *Ae. albopictus* populations in combination with educational campaigns have shown significant mosquito population reductions [20,25].

Other novel control methods, such as the release of genetically modified mosquitoes or irradiated males (sterile insect technique), the modification of mosquitoes’ midgut flora (paratransgenesis), or the implementation of different biological control methods like the release of predatory copepods, or the employment of entomopathogenic bacteria (i.e., *Wolbachia pipietensis*) and fungi (i.e., *Bauveria bassiana*) are promising tools which could be exploited in the future.

Trapping is extensively used for mosquito monitoring, but its capacity for population control is still not well studied, and in particular there is very limited research on massive lethal trapping for *Aedes* sp. Different types of lethal ovitraps for *Ae. aegypti* control in field trials, however, have shown significant reduction in larval and adult indexes in Brazil [26] and dengue cases in Malaysia [27].

The NESCOTIGER project aimed to design and assess a novel control strategy against *Ae. albopictus* in urban residential areas of Eastern Spain. An integrated control program was implemented in the study area based on three main pillars: the citizen-based mass trapping deployment of control tools with different modes of action, the treatment of breeding sites in public areas (SCB) with the combination of a long-lasting insecticide paint and entomopathogenic bacteria mixture, and an educational campaign addressed to residents through workshops, leaflets, and social media. Other studies previously published as part of the NESCOTIGER project focused on complementary aspects of this strategy such as knowledge, attitude and practice, and breeding site surveys. Their key findings are also included in the current manuscript to enhance understanding and to obtain a bigger picture of the project’s obtained results.

## 2. Materials and Methods

### 2.1. Study Site

**Figure 1:**
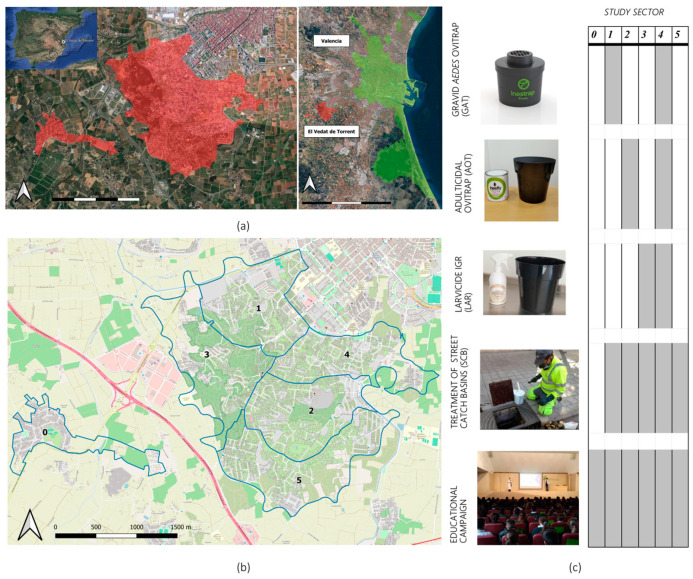
NESCOTIGER’s study area and sectorization. (**a**) Location of the residential area of El Vedat de Torrent (red) and Valencia (green). (**b**) Sectorization of the residential area of El Vedat de Torrent; blue lines = boundaries of the study sectors (0 to 5; *n* = 6). (**c**) Trapping tools and other interventions deployed in the different sectors.

The field trial was conducted in the residential area of El Vedat de Torrent (39°25′25″ N 0°29′35″ W), located 9 km from the city of Valencia and near the Mediterranean coast of East Spain (Figure 1). This area occupies a small mountain with a surface of 5.86 km^2^ and sits 142 m asl. Pine woodlands are integrated into the site, and agriculture fields also surround the area. The main climate data recorded during the study period (June to October 2022) were 40.7 °C, 12.6 °C, and 25.6 °C for the maximum, minimum, and average temperatures, respectively. Total cumulative rainfall was 66.1 mm, but precipitation was mostly concentrated in October (31.7 mm) (Valencia Airport meteorological station, AEMET).

The first construction in the region dates to 1890. The area then experienced intense urbanization during the sixties and seventies, and now there are 3173 registered houses (detached and semi-detached), with an estimated population of 12,500 inhabitants. This residential area has public water services and is characterized by large single-family homes (parcel size: $\bar{x}$ = 642 m^2^; $\tilde{x}$ = 591 m^2^; Mo = 591 m^2^; min = 124 m^2^; max = 2133 m^2^; SD = 390 m^2^), each usually having a private swimming pool and garden areas.

Following its first detection in 2016, *Ae. albopictus* has become firmly established in the area [28], and the municipality conducts larviciding in SCBs as a reactive intervention due to resident complaints.

### 2.2. Study Design

**Figure 2:**
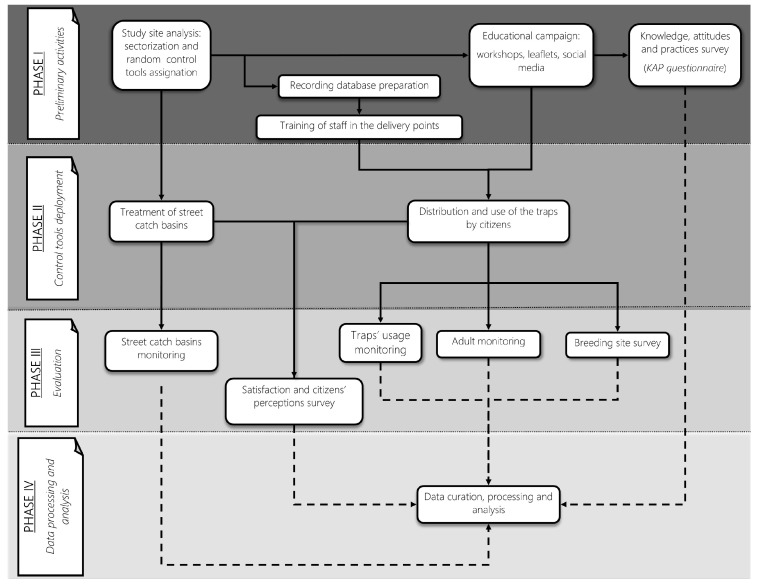
The NESCOTIGER project workflow chart, showing the different activities of each research phase (I–IV). Continuous lines represent related tasks of the project, and discontinuous lines represent data from a particular activity that will later be used for data curation and analysis.

The study was a non-randomized controlled trial with a treatment-control design aimed at evaluating the effectiveness of four different trapping interventions in controlling *Ae. albopictus* populations. From an entomological perspective, a descriptive longitudinal study was conducted, utilizing various entomological indicators measured in the different treatment and control sectors. The project was divided into four phases (Figure 2), which are briefly described below.

#### 2.2.1. Phase I: Preliminary Activities

##### Study Site Analysis—Sectorization and Random Control Tools Assignation

Five study sectors were delimited based on the criteria of having similar study surface areas and the number of houses per sector. Afterwards, each sector was randomly assigned to one of the five combinations of control interventions (Figure 1). An additional sector, separated from the study area, was selected as the initial null control, which, based on data results and field inspections during Phase II, was later discarded due to major ecological differences with the rest of the study area. Nevertheless, as it will be later discussed, Gravid *Aedes* traps (GAT, described below) did not show any capture efficiency, for which sector 1 was regarded as a placebo/null control area and will be referred to as such during the rest of the manuscript.

##### Distribution Database Preparation and Training of Delivery Points Staff

A database was created with Microsoft Excel employing public land registry data [29] that included houses’ addresses and parcel sizes. Households were assigned an ID and a sector number based on the sectorization of the territory, which determined the type of traps to be used and their number (1 to 4) according to the size of the plot. The number of units per parcel’s surface was defined for each sector based on the size quartile to which the household belonged. The database operation was taught to each of the delivery point staff in private one-to-one sessions by members of the research team.

##### Educational Campaign

Residents were informed through the city council and neighborhood associations about the NESCOTIGER project. Several talks were conducted before the mosquito season, each including a brief description of the project and basic concepts of the *Ae. albopictus* biology and control. The research team invited residents to join the study. Children from primary schools linked to El Vedat participated in workshops dealing with these topics. The research team designed and executed these activities, tailoring them to the project’s objectives.

Leaflets, social networks, web pages, and WhatsApp were the other channels used for spreading information about the project and cultural practices for *Ae. albopictus* control, combined to form a complete educational campaign that supported trap use among participants. Continuous communication with the participants was set by email, telephone, WhatsApp, and the neighborhood associations.

##### Knowledge, Attitude, and Practices Survey

A knowledge, attitude, and practices (KAP) survey was carried out among primary school children and adults living in the study area as described elsewhere [30]. A general score [0–10] was obtained for each respondent based on correct knowledge questions. A descriptive bar-diagram analysis and a Wilcoxon signed-rank test was conducted comparing median scores between both adults and children respondents. Identified breeding sites by children and adults was compared through odds-ratio and Fisher’s exact *t*-tests and graphically represented as a bar diagram. 

#### 2.2.2. Phase II: Control Tools Deployment

##### Treatment of Street Catch Basins

Before the distribution of the trapping tools (March and April 2022), cartographic materials were generated based on the available public data on the location of SCB in public areas. A team composed of two vector control technicians systematically treated with insecticide paint (Inesfly 5A IGR NG, Inesfly Corporation S.L., Paiporta, Spain) and a combination of *Bti* and *Lysinbacillus sphaericus* (*Lsph*) (VECTOMAX, Valent Biosciences, Libertyville, IL, USA) all the SCB located in public areas based on the available charts, as well as any other ones found during the interventions. The insecticide paint was applied to SCB with a roller on the aerial surface of the chamber while making no contact with the contained water. A commercial black dye for water-borne paints was added to draw females’ attraction to these oviposition sites. This dye was added to the paint applied in the street catch basins to provide an attractive stimulus for oviposition and hence favour the effectiveness of this control measure deployed in public areas [31]. During phase II (May to October 2022), residents’ complaints that led to reactive treatments with VECTOMAX were carried in SCB within a 150 m buffer radius from the original report site. Total treated SCB per hectare is represented as a bar diagram. 

##### Trap Distribution and Use by Citizens

Traps were given to citizens between April and July 2022 by distribution staff in several delivery points managed by neighborhood associations and the municipality. Residents who voluntarily decided to join the project and receive the traps were provided with informed consent for their signature. Participation per sector was evaluated as the percentage of households included in each sector divided by the number of registered residences in the area, and is presented as a bar diagram per sector. The traps delivered were recorded in the database. Total traps per household and ha in each sector were also represented as bar diagrams. Printed instructions about the use, placing, and maintenance of the traps were given, and short informative videos were available on the NESCOTIGER project website. Door-to-door visits to provide project information and deliver traps to residents were also conducted by the research team, but these were focused solely on the evaluation zone defined in each sector. 

#### 2.2.3. Phase III: Evaluation

##### Monitoring of Street Catch Basins

Drainage infrastructures in public areas were randomly inspected in two rounds during June and September 2022, which marked a dry and rainy period, respectively. Catch basins were sampled by systematic dipping (around 300 mL/SCB). Water samples were divided into two 150 mL aliquots. Larvae contained in one of the aliquots was reared in the laboratory until the complete adult emergence for species identification. Immature stages present in the other aliquot were fixed with hot water and ethanol 70% for morphological species identification and counting with the aid of a stereomicroscope. Larvae presence and adult emergence was compared between identified species both inferentially (Wilcoxon signed-rank test) and descriptively (bar-diagrams). 

##### Monitoring of Trap Use

Trap placement and maintenance were assessed in June and September through inspections at randomly selected houses that were not informed in advance. Traps deployed in the households were inspected by the research team and evaluated in terms of being properly located according to the given instructions (exterior, shaded area, and close to vegetation) and filling with water. Percentage of properly used traps per sector and inspection is presented as a bar diagram. Positive breeding in the water contained in the adulticidal ovitraps and the larvicide-treated containers was registered, and water samples were collected for laboratory rearing until adult emergence observation and species identification. Additionally, citizens who used the sticky ovitraps (GAT) were asked to return the used sticky adhesives monthly. Sticky cards were processed, and the number, sex, and species of captured mosquitoes were registered. A descriptive analysis of total captured individuals was conducted, while an inferential (Wilcoxon signed-rank test) and descriptive (bar diagram) comparison between total male and female caught mosquitoes was conducted. 

##### Breeding Sites Survey

A larval and pupal survey was carried out between June and August 2022 at randomly selected households of each of the study sectors and the null control. Exterior areas (i.e., gardens) were inspected for real and potential mosquito breeding sites, which were recorded. Water samples from breeding sites with preimaginal mosquitoes were collected, processed, and classified under laboratory conditions for quantification and species classification. A complete description of the methodology can be found in another publication on the NESCOTIGER project [19]. Total larvae and pupae found per breeding site type are represented as a bar diagram. A graphical representation of Breteau Indices (BI) per study sites, as described by [19], is included, while a Kruskal Wallis *t*-test addressing the effect of study site over BI was conducted. The effect of total deployed lethal ovitraps (LOT, described below) in a 150 m radius from each inspected household over the total abundance of larvae per household was determined through a general lineal model (glm). Results from the glm are presented as a scatter-plot diagram. 

##### Adult Monitoring

An adult entomological survey was conducted based on a citizen science perspective. Two BG-Sentinel 2 traps (Biogents A.G., Regensburg, Germany) per study sector (n = 12 total adult capturing traps), provided with a chemical lure (BG-Lure; Biogents A.G., Regensburg, Germany), were placed in randomly selected households. Researchers positioned these surveillance devices in the gardens connected to houses’ power outlets and individually trained participants in the handling of the tools. Citizens were periodically reminded to operate traps for 48 h every two weeks from June to October 2022 in a simultaneous way across all the sectors. Collection bags were placed in freezers until collected by the research team, who then sorted the captured individuals by species and sex in the laboratory. A comparison of total median captured female *Ae. albopictus* between sectors was conducted through a series of Wilcoxon signed-rank test comparing the placebo sector (GAT) with the other study areas and represented as a boxplot chart. The effect of trap deployment over female *Ae. albopictus’* abundance was evaluated through a generalized linear mixed model (glmm) addressing the effect of the abundance of each of the deployed traps and SCB in a 150 m radius from each inspected household. Study sector was set as a random-effect variable. A general lineal model (glm) was also conducted evaluating the effect of deployed adulticidal traps (AOT, described below) in a 150 m buffer radius from surveillance devices over respective captured female *Ae. albopictus*. Results from the glm are presented as a scatter plot. Additionally, a 4 × 4 matrix was generated, representing the mean number of units for each trapping tool in a 150 m buffer radius from surveillance devices in each sector (rows, sectors; columns, trap device), while a vector of median captures of female *Ae. albopictus* per sector and date was created (n = 11 vectors; each containing four values). Each matrix was individually solved, and a vector of the estimated effect of trap abundance in 150 m over mosquito abundance was obtained for each date. Individual vectors were grouped in a single data frame per date and presented as a scatter plot. 

##### Satisfaction and Residents’ Perception Survey

At the end of the mosquito season (October 2022), a survey was conducted to gather residents’ perceptions and satisfaction with the NESCOTIGER project. A structured questionnaire with 21 questions prepared with Google Forms was distributed among residents in El Vedat de Torrent mainly through mass emailing by the principal neighborhood association that collaborated with the project. The questionnaire included some satisfaction questions concerning the information related to the project, the attention received by the research team, the delivery of the traps, and general satisfaction with the project; other groups of questions aimed to evaluate practices related to additional mosquito control activities carried out during the project period as well as trap maintenance. The last group of questions dealt with the perceptions of citizens concerning the number of bites and densities of mosquitoes, their willingness to participate in a similar project, and any perceived adverse effects. The complete questionnaire is available in Appendix A. Results are presented as bar diagrams, showing the percentage of positive answers per each question, grouped by satisfaction, practices and perception questions. Additionally, a comparison between sectors was conducted concerning the perception of mosquito abundance between the study period and previous seasons through odds ratio and Fisher’s exact *t*-tests. 

#### 2.2.4. Phase IV: Data Processing and Analysis

Data curation and spatial, descriptive, and inferential analysis were conducted using the QGIS (QGIS Development Team (2024), version 3.36.1 for Windows) and Rstudio (The R Foundation for Statistical Computing (2024), version 4.3.3 for Windows) software, and graphics were created using the software GraphPad Prism (GraphPad Software (2024), version 10.3.1 for Windows). Statistical difference for the conducted inferential test was established for values of *p* ≤ 0.05, while a slight statistical difference was accepted for *p* ≤ 0.10. Homoscedasticity was evaluated in each analysis with a Bartlett test (‘bartlett()’ function from base R), while a normal data distribution was studied using qqplot graphs and Shapiro-Wilk tests (‘shapiro.test()’ from base R). A KAP survey and Aedic indices data were included in previous published research [19,31]. Microsoft Excel (Microsoft Team (2019), version 1808 for Windows) was used for creating the database for plots and trap management.

### 2.3. Traps and Control Tools Assigned to the Study Sectors

The GAT used was a black plastic container with a cover and a capture chamber employing a sticky band. Some vegetal matter was added to tap water to enhance its attractiveness for *Aedes* mosquitoes’ oviposition. This trap was assigned to sector 1, and based on observed null captures (previously discussed) is regarded during the present research as a placebo/positive control tool.

For this study, adulticidal ovitraps (AOT) were black plastic containers (diameter 20 cm, 4.2 L) that had their internal walls coated with a black-tinted insecticide paint (INESFLY 5A IGR NG, Inesfly Corporation S.L., Paiporta, Spain) with a composition made up of alphacypermethrin 0.7%, d-allethrin 1.0%, and pyriproxyfen 0.063%. The paint dose was approximately 35 mL per bucket and was equivalent to 10 m^2^/L. This treated container was filled with tap water for oviposition attraction. This trap was assigned to sector 2.

The larvicide (LAR) (INESFLY LARVA IGR, Inesfly Corporation S.L., Paiporta, Spain) used was a spray formulation (500 mL spray bottle) for surface treatment containing 0.2% pyriproxyfen. It was applied to dry surfaces that could become mosquito breeding sites, e.g., plant pot plates, drains, fountains, and other water-holding containers. The larvicide was also applied to black plastic buckets (diameter 20 cm, 4.2 L) provided by the project for transforming them into LOT. These treated containers were filled with tap water for oviposition attraction. Larvicide application to objects and black plastic containers was conducted by residents in their households. This control tool was assigned to sector 3.

Sector 4 received a combination of the three trapping models to explore a pull-push strategy.

### 2.4. Ethical Aspects

Inclusion in the project was voluntary, and participants signed a written informed consent when collecting the traps. Confidentiality of the gathered information and data protection were ensured through the application of national regulations. Traps and other project-related materials were provided at no cost to the residents.

## 3. Results

### 3.1. Knowledge, Attitude, and Practices Survey

**Figure 3:**
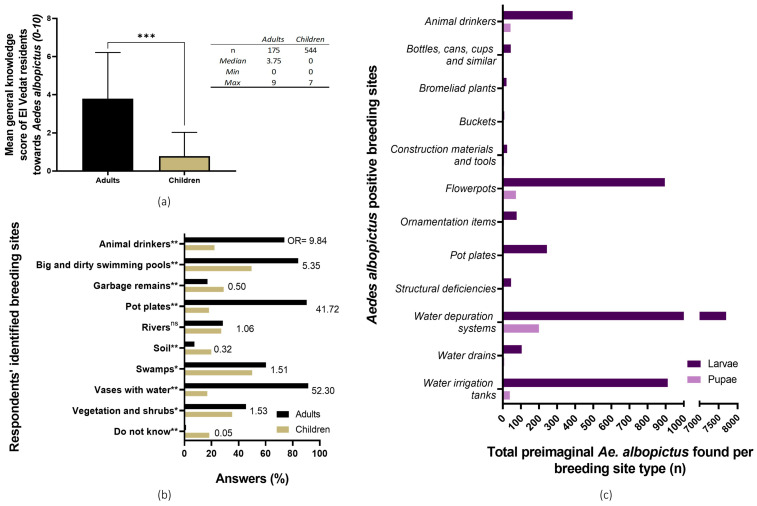
Citizens’ knowledge of *Aedes albopictus*’ biology and breeding sites in El Vedat de Torrent. (**a**) Children and adults’ average score of basic knowledge about *Ae. albopictus*’ biology. ***, *p* < 0.001, Wilcoxon signed-rank non-parametric variance test. (**b**) Existing knowledge of *Ae. albopictus*’ breeding sites from the KAP survey; breeding sites identified by adult (black bars) and children (brown bars) respondents. The probability of selection for each breeding site between study groups (adults against children) was compared through an odds ratio and Fisher’s exact *t*-test. (**c**) Identified positive breeding sites (purple = larvae; pink = pupae) during the larval survey. (OR = odds ratio; ns = no significant differences, *p* < 0.05; *, *p* < 0.05; ** = *p* <0.01).

Both adults and children’s general knowledge of *Ae. albopictus*’ biology was considered to be very low to none, with grown-ups having a better general understanding of this mosquito species than primary school students (Wilcoxon signed-rank test, *p* < 0.0001; adults’ general knowledge score: 3.75/10; children’s: 0/10). Concerning *Ae. albopictus*’ breeding sites identification by citizens, in general, children underperformed in comparison to adults. Many respondents from both age groups misidentified “big and dirty swimming pools” as *Ae. albopictus* tiger mosquito’s preimaginal foci (Figure 3). For a more in-depth description of the KAP survey results, the Alarcón-Elbal et al. paper can be consulted [30].

### 3.2. Trap Deployment and Monitoring

**Figure 4:**
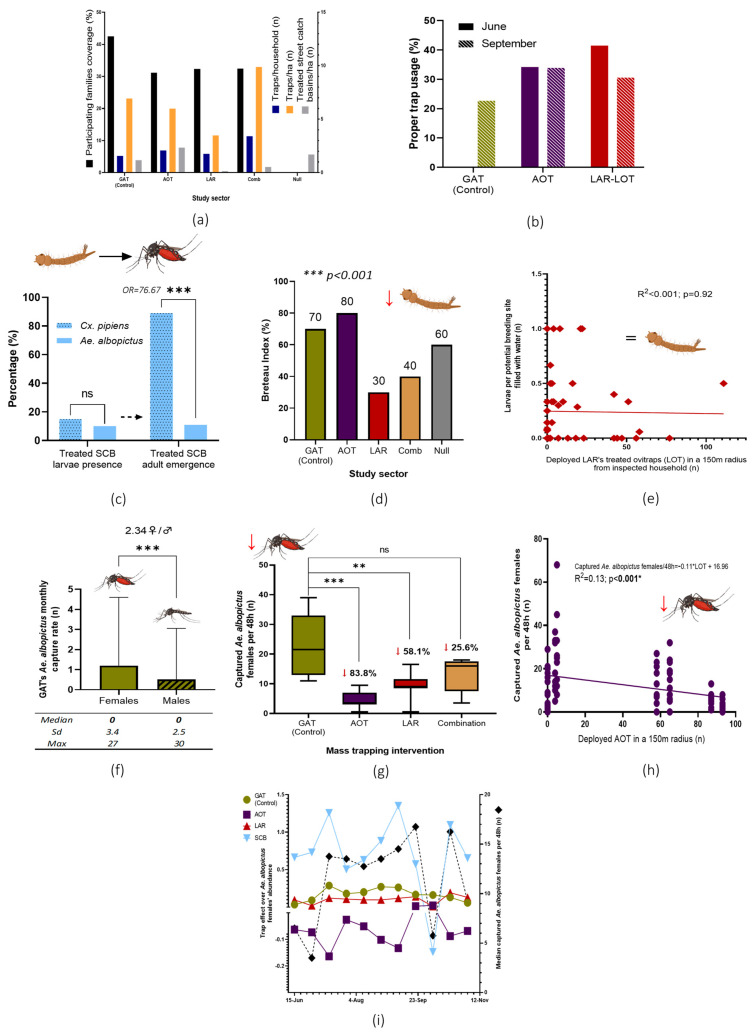
Principal results and analysis of the NESCOTIGER project. (**a**) Results of participants’ trap deployment and treated SCB in the study sectors. (**b**) Evaluation of trap usage by citizens: correct location and maintenance. (**c**) SCB positivity rate in mosquito immature stages and adults’ emergence from sampled water; odds ratio and Fisher’s exact *t*-test (ns = no statistical significance; *** *p* < 0.0001). (**d**) BI was obtained for all sectors in the breeding site survey. Differences among sectors were confirmed through a non-parametric Kruskal–Wallis variance *t*-test (*p* < 0.001). (**e**) LOT deployed units’ effect over larvae abundance in a 150-m radius from inspected households analyzed with a lineal regression model. (**f**) Mean monthly captures of *Ae. albopictus* by GAT (*** = *p* < 0.0001 Wilcoxon signed-rank test). (**g**) *Ae. albopictus* female captures in the study sectors with BG-Sentinel 2 traps with percentage reductions (ns = no statistical significance; ** = *p* < 0.001; *** = *p* < 0.0001). (**h**) Lineal regression model analysis (glm) of *Ae. albopictus* females and AOT units deployed in a 150-m radius. (**i**) Matrix analysis of the control effect over *Ae. albopictus* populations exerted by the control tools during the study period. Downward red arrows represent general statistically significant adult or larvae mosquito populations. SCB, street-catch basins treatment with insecticide paint and entomopathogenic bacteria mixture; GAT, gravid *Aedes* trap; AOT, adulticidal ovitraps; LAR, larvicide; LOT, larvicide ovitraps, Comb, combination of SCB, GAT, AOT and LAR interventions; Null, no control intervention.

A total of 2884 traps were handed to 1028 participating families in six delivery points, which represented a general coverage of 32.4% of the households in the study area (GAT, 42.5%; AOT, 31.1%; LAR, 32.3%; combination, 32.4%) (Figure 4a). Deployment rates in sectors 1 to 3 were similar (2.23 GAT, 2.73 AOT, and 2.33 LOT per household) but higher in sector 4 (4.24) as expected due to the trap’s combination design.

A mean estimate of 6.57 traps per hectare was achieved among all sectors where traps were deployed. This density was noticeably lower and higher for sectors 3 and 4 (3.48 traps/ha and 9.89 traps/ha, respectively) while being similar in the other sectors (S1 = 6.94/ha and S2 = 5.99/ha).

A total of 69 randomly selected households were inspected during two study periods in June (n = 25) and September (n = 44) 2022. Inspected traps showed low proper usage percentages, having registered an estimated average of 37.85% and 29.07% of properly maintained and placed traps in June and September 2022, respectively (Figure 4b).

No larvae or pupae-positive AOT were found in June among the 31 of 44 that contained water, and one of the 35 traps from 56 containing water was found positive in September. Positivity of the LOT was 30.0% (12/40) and 36.0% (9/25) in the June and September inspections, respectively. Only two *Ae albopictus* adults emerged from two of the positive water samples collected from LOT inspected in June and September.

Sticky adhesive cards of the GAT captured more *Ae. albopictus* females than males (2.34; Mass-Whitney test, *p* < 0.0001). Nevertheless, out of the 245 inspected cards, a median value of zero monthly captures of *Ae. albopictus* implied a null effect on mosquito populations (Figure 4f). Based on these results, GAT devices deployed in sector 1 were regarded during the present research as a placebo/positive control tool.

### 3.3. Treatment and Monitoring of SCB

A total of 695 SCB were identified and treated in the study area during March 2022. A follow-up inspection of 100 randomly selected SCBs conducted five months after treatment revealed that only 1.1% of the total evaluated SCBs showed *Ae. albopictus* adult emergence (36% contained water and 10.00% were larval positive, with an estimate of 10.98% emergence among the positive SCB). In contrast, *Culex pipiens* (Linnaeus, 1758) emergence was observed in 13.35% of all the inspected SCB (15.00% positive SCB, with an emergence of 89.02% in positive SCB. No statistical differences were observed between *Ae. albopictus* and *Cx. pipiens* larvae presence in the evaluated SCB (Fisher’s exact *t*-test; *p* = 0.39). In contrast, *Ae. albopictus* emergence from positive SCB was lower than that of *Cx. pipiens* (OR = 76.67; Fisher’s exact *t*-test, *p* < 0.0001) (Figure 4c).

### 3.4. Breeding Sites Survey

A total of 33 out of 1143 inspected breeding sites in residential private areas were found positive for *Ae. albopictus* larvae or pupae during household inspections. Based on obtained field data, several entomological indices were established (house index (HI) 40 positive houses per 100 inspected residences; container index (CI), 2.89 positive breeding sites per 100 inspected; pupal index (PI), 633 pupae per 100 inspected houses; BI, 55 positive breeding sites per 100 inspected households). Statistical differences concerning the BI were observed among the different study sectors (Kruskal–Wallis, *p* < 0.001). Areas where the larvicide was implemented showed the lowest values (Figure 4). Nevertheless, no lineal effect was observed between traps’ abundance in 150 m from the inspected house over the abundance of larvae per breeding site with water per house (glm; larvae per breeding site with water ~ GAT + AOT + LOT + SCB; adjusted R^2^ = 0.0018, *p* = 0.32). The isolated effect of the larvicide in a 150-m radius from inspected houses was also not associated with larva abundance (Figure 4d).

While the most commonly identified breeding sites were pot plates, flowerpots, and buckets (38.1%, 28.6%, and 14.3%, respectively), the total number of both larvae and pupae was similar or even lower than other less common and cryptic foci. In this sense, a single massively infested water depuration system showed the highest *Ae. albopictus* abundance, having presented an estimate of 7715 larvae and 205 pupae (Figure 3c). For a more in-depth description of the general results of the breeding sites survey, please refer to previously published research [19].

### 3.5. Adult Monitoring

A total of 2094 adult mosquitoes belonging to three species were captured and identified through monitoring with the BG-Sentinel 2 traps for 11-fortnight rounds: 1674 *Ae. albopictus* (1160 females, 514 males), 363 *Cx. pipiens* (246 females, 117 males), and 57 *Culiseta longiareolata* (Macquart, 1838) (37 females, 20 males). Hereafter, the data only reflect captures concerning female *Ae. albopictus*.

As the GAT was previously observed to not have a controlling effect over *Ae. albopictus* populations, sector 1 was set as the placebo/positive control area. A statistically significant reduction of mosquito abundance was observed through a Wilcoxon signed-rank test in sectors with massively deployed AOT (83.8% reduction; *p* < 0.0001) and larvicide-LOT units (58.1%; *p* < 0.001) in comparison with the placebo sector 1. The combined effect of the different trap models (sector 4) also showed a mosquito reduction with near statistical significance (25.6% reduction; *p* = 0.06). An increase in the deployed AOT per 150-m radius from monitoring devices showed a reduction in mosquito abundance (glm; captured *Ae. albopictus* per 48 h~16.96 −0.11AOT; R^2^ = 0.13, *p* < 0.001) (Figure 4h). When accounting for all the existing traps per 150 m, a better model fit is observed (Table 1). This AOT effect was consistent over time, as observed in the matrix analysis (Figure 4i). Based on these data, an estimate reduction of 8.56% in female *Ae. albopictus* abundance for each 10 deployed AOT in a 150 m radius was estimated based on the following equation:

*Ae. albopictus* reduction per 10 AOT = (((−0.09 individuals/AOT) × (10 × AOT))/11.57) × 100 = −8.56%

### 3.6. Residents’ Satisfaction and Perception Survey

**Figure 5:**
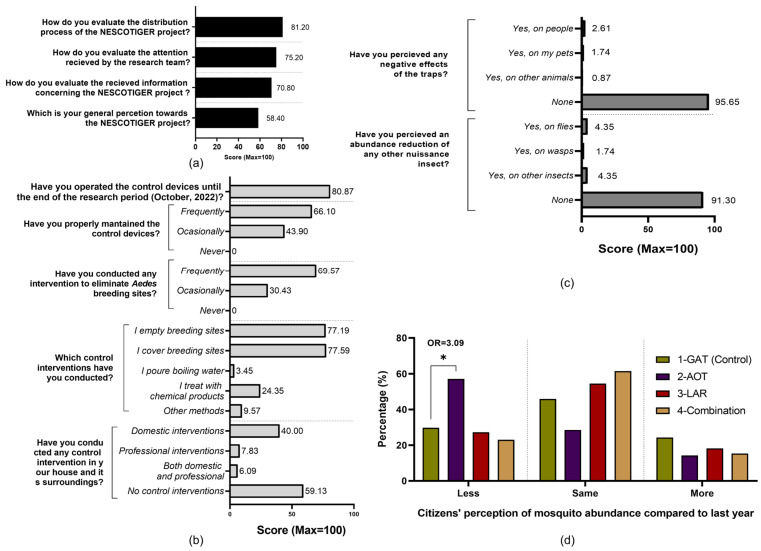
Residents’ satisfaction and perception survey results. (**a**) Satisfaction of respondents participating in the NESCOTIGER project. (**b**) Practices of respondents participating in the NESCOTIGER project towards *Ae. albopictus* control activities. (**c**) Residents’ perceptions about the side effects of the traps. (**d**) Evaluation of participants’ perceived mosquito abundance variation between 2021 and 2022 (less, same, or more mosquito abundance in 2022 than in 2021). The data were grouped by study sector (* = Fisher’s *t*-test *p*-value < 0.05; OR = odds ratio).

A total of 116 residents completed the satisfaction and perception survey, 50.65% being male (n = 59), 48.48% female (n = 56), and 0.87% undisclosed (n = 1). Respondents’ ages ranged from 26 to 80 years old (mean = 58.84, median = 60.5, SD = 13.33). Most surveyed citizens were originally from Spain (n = 114/116), while one Italian and one Cuban also filled out the questionnaire. Participants’ general satisfaction towards the project and attitudes and practices concerning *Ae. albopictus* scored 58.4% but specific aspects like the distribution process were deemed as largely positive (81.2%) (Figure 5a).

Participants reported frequent and extensive implementation of cultural control methods related to breeding site management, and all these practices were carried out by the residents with limited professional interventions being reported. Traps were in service during the whole season and frequently maintained. Almost all respondents (95.65%) did not perceive adverse effects potentially linked to the traps or any impacts on other insect populations (91.30%) (Figure 5b).

Data from the AOT sector revealed a statistically significant reduction in the perceived reduction of mosquitoes in comparison with the control area and the previous year (*p* < 0.05; OR = 3.09). The sum of lower and same mosquito abundance reported by residents in sector 2 (85.7%) was higher than the other sectors, especially in comparison to the control (40.0%) (Figure 5d).

## 4. Discussion

Integrated vector management highlights the importance of understanding vector species’ biology and local environmental factors to develop targeted strategies, tools, and actions for controlling vector populations and reducing disease burden [32]. The domestic behavior of mosquitoes, particularly synanthropic *Aedes* species, poses significant challenges to the effective implementation of control interventions [33].

The NESCOTIGER project represents a pioneering effort in Europe, showcasing a comprehensive and innovative approach to controlling *Ae. albopictus* populations. This multifaceted strategy, combining mass trapping, larvicidal measures, and citizen science, sets a new standard for vector management in urban residential areas, highlighting the potential for scalable and sustainable mosquito control solutions across different socio-ecological contexts. Additionally, the large-scale field trial, involving over 1000 families and nearly 3000 traps, provides extensive data on mosquito population dynamics and control efficacy. Moreover, it builds on relevant previous results [19,31], which, combined with the latest findings presented in this study, make this project a benchmark for continued progress on mosquito control at the European level.

### 4.1. Treated Public Street-Catch Basins Played a Negligible Role as Ae. albopictus Breeding Sites in Comparison with Private Peridomiciles Within a Residential Area

The existing literature has identified SCB as a common breeding site for several mosquito species [34]. In the study area, both *Ae. albopictus* and *Cx. pipiens* were frequently found in these structures, with colonization rates of 10.00% and 15.00%, respectively, 20 weeks after treatment with the insecticide paint. As reported by Pettit et al. [35] under field conditions, alphacypermethrin-treated breeding sites can prevent colonization by certain *Culex* and *Aedes* species for up to 11 and 20 weeks, respectively. However, the long-term efficacy of this insecticide paint in inhibiting *Ae. albopictus* colonization in SCB under real-world conditions requires further investigation. Additionally, SCBs were treated with a combined application of the microbial control agents *Bti* and *Lsph*. While *Bti* presents a rapid larvicidal effect within 24 h after treatment and an estimated four-week residual effect in treated sites, *Lsph* extends the larvicide effect due to the recirculation of the bacteria in dead larvae, especially of *Culex* spp [22].

Nevertheless, *Ae. albopictus* collected from treated basins exhibited significantly lower adult emergence rates than *Cx. pipiens* in emergence assays (10.98% vs. 89.02%; OR = 76.67; Fisher’s exact test, *p* < 0.0001) (Figure 4c). Consequently, an estimated 389 adult mosquitoes emerged weekly from SCB within the study area (0.56 adults per week × 695 basins). In contrast, during the same inspection period, 385 pupae were found in 60 households surveyed. Given that the study area comprised 3173 registered residences, and assuming complete pupal survival, an estimated 20,360 adults emerged weekly from private properties. The low yield of adult generation from the treated SCB derived from the water presence, larval positivity and adult emergence suggests that SCB played a negligible role in sustaining *Ae. albopictus* populations. It is important to highlight that public control investments against *Ae. albopictus*, which can be substantial [36], are primarily directed at public breeding sites and addressing only a fraction of the potential breeding habitats since most of these habitats are located within residential areas. In addition to using biocides for mosquito control in these structures, modifying the sand sewer drain type to limit the mosquitoes’ reproduction in artificial reservoirs has also been proposed. This strategy, successfully implemented in Catalonia (Spain), has proven effective and ultimately represents a more sustainable long-term solution [37].

### 4.2. Mass Trapping Community-Based Interventions, Which Can Access Private Areas, May Be Jeopardized by a Lack of Citizen Participation and Tools Misused

According to this finding, interventions targeting *Ae. albopictus* breeding sites in residential areas have become a major priority [38,39]. Even though door-to-door strategies focused on source reduction in private areas are an interesting alternative, such an approach entails a considerable increase in expenses and organizational effort in comparison to routine interventions conducted in public areas with similar results, important factors that need to be taken into consideration [40]. On the other hand, the concept of community-based mass trapping control interventions for synanthropic *Aedes* mosquitoes in urban areas may drastically lower the human-associated costs of intervention programs [41,42]. Regardless of the exact approach, citizens’ participation and interest are considered essential to the success of any community-based intervention. In the case of our study area, it should be noted that only 32.4% of households took part in the research (Figure 4a), and less than 40% to 30% of the traps were properly used (Figure 4b). Interestingly, during the perception survey, all responding participants affirmed to have properly maintained the control tools either periodically or occasionally (Figure 5b). Nevertheless, reduction effects of both preimaginal and adult *Ae. albopictus* populations were observed in the field. A description of the effectiveness of each of the deployed control tools—GAT, LAR, and AOT appears below.

### 4.3. Gravid Aedes Traps Showed No Effect over Adult Female Ae. albopictus Populations

Sector 1, where only GAT devices were distributed, was regarded as the placebo/positive control area during the field assay, as a null median capture rate was observed during the inspection of the traps’ sticky cards. Nevertheless, an increase of mean captured females over males was observed in the adhesive sheets (Figure 4f), as evidenced in previous field assays where gravid females seeking oviposition sites were more attracted to GAT than other female gonotrophic stages or males [43]. Previously published research has shown the potential employment of commercial GAT in synanthropic *Aedes* management, as exemplified by the population suppression of *Ae. albopictus* for over six months through a massive deployment of Biogents’ BG-GAT model (Biogents A.G., Regensburg, Germany), though this instance also used other extensive control activities in an island in the Maldives [44]. In this sense, it should be noted that the employed GAT has been modified by the manufacturer in its design since the NESCOTIGER project, having shown promising results in multiple independent field assays, evidenced by a 2.46 to 4.34 proportional increase capture efficiency of *Ae. albopictus* in comparison with the BG-GAT and a capture rate between 1.25 to 3.79 trapped individuals per sampling day in a similar environment to that of the current research [43]. As such, the effectiveness of this novel GAT model in future mass trapping programs should be evaluated.

### 4.4. Larvicide Spraying of Identified Private Breeding Sites by Participating Citizens Showed a Reduction of Both Larvae and Adult Abundance

In contrast, the general larvae abundance was reduced in areas where the larvicide spray was massively distributed to citizens (Sectors 3 and 4), as shown through the reduction of the BI obtained during the Aedic indices assay (Figure 4d) [19]. This strategy was based on self-pulverization by citizens of breeding sites in their residential areas (scuppers, drains, pot plates, etc.). This special formulation releases pyriproxyfen once the treated object is filled with water, inhibiting adult emergence, as observed by Tilak et al. when assessing this formulation for controlling *Ae. aegypti* breeding in desert coolers [45]. Additionally, citizens were handed empty black containers for their pulverization and deployment in their backyards as active trapping devices for mosquito larvae (LOT). No lineal correlation was observed between the number of deployed containers and the number of larvae in a 150-m radius (Figure 4e) or in the number of adults, as evidenced by the general mixed model (Table 1), even though a general reduction of the median number of captured adult female *Ae. albopictus* was observed when compared to the GAT control area (Figure 4g). It should also be noted that this strategy relies on the knowledge of citizens regarding *Ae. albopictus*, which was shown to be low for both adults and especially children in the study area (Figure 3a) [19]. Based on the data obtained from previous research [19,31], it can be argued that citizens did not correctly identify all the breeding sites in their residential areas (Figure 3), which in fact could have greatly reduced the effectiveness of this strategy. As such, the larvicide may have exerted a qualitative reduction effect over immature mosquito populations, which may have been reduced by residents’ misidentification of potential *Ae. albopictus* breeding sites.

### 4.5. Adulticidal Ovitraps Mass Distribution Showed the Highest Mosquito Abundance Reduction

The greatest *Ae. albopictus* adult female population reduction was observed in Sector 2, where AOT were massively distributed to citizens (Figure 4d). A lineal inverse correlation was observed between the number of deployed traps and the density of adult females per 150-m radius (Figure 4h), while an estimated reduction of more than 8% BG-Sentinel captured females in 48 h per 10 deployed AOT was estimated through the mixed general lineal model (Table 1). Notably, AOT were coated with the same insecticidal paint as employed for the treatment of SCB, containing alphacypermethrin, d-allethrin, and pyriproxyfen, potentially causing the mortality of both larvae and adults, as well as sterilizing females. Previous laboratory assays employing a similar formulation produced by the same manufacturer showed that insecticide-coated oviposition containers caused a considerable adulticidal (81%) and sterilization effect (98.7%) on gravid *Ae. aegypti* females while also completely inhibiting preimaginal breeding [46], the latter effect being also observed in the field deployed AOT in the current study area. While alphacypermethrin pulverizations of breeding sites may have a residual inhibition effect over mosquitoes for several weeks [35], the microencapsulation of biocides and their application in this insecticide paint has been shown to increase treatment persistence against *Aedes* sp. for up to 36 months [47]. In this sense, the measured mosquito reduction effect of AOT was consistent over the study period (Figure 4i).

From the insecticide resistance perspective, the selective pressure with one insecticide class is known to lead to the replacement of susceptible by resistant populations. The paint applied in the AOT contained a combination of pyrethroids and pyriproxyfen. The effects exerted by this insect growth regulator tends to mitigate the offspring of pyrethroid resistant individuals from pyrethroid resistant females as observed by Cárdenas et al. with a similar insecticide coating and *Ae. aegypti* [46].

Additionally, most surveyed citizens with AOTs (Sector 2) reported a perceived reduction of mosquito abundance in comparison to previous years, with such perceptions differing greatly from the responses given by participants in the control area (Figure 5d). The observed association of neighbors’ perceptions and entomological parameters was also found in other studies related to *Ae. albopictus* control [22,40].

### 4.6. Citizens’ Knowledge Towards Ae. albopictus’ Biology Is Regarded as a Major Milestone Towards This Mosquito Mass Trapping Control in Residential Areas

While most previous research delving into mass trapping strategies has focused on *Ae. aegypti*, less attention has been paid to *Ae. albopictus* [41]. Nevertheless, given the major ecological and ethological differences found between both vectors [48], mass trapping control campaigns must exploit available tools in divergent ways. As an example, recent research conducted in Cabo Verde tested the employment of similar insecticide paint formulations for indoor house painting addressed to the control of *Ae. aegypti* [49], using their paint indoors instead of for treating SCB or AOT. Overall, NESCOTIGER’s results point to the effectiveness of *Ae. albopictus* management in residential areas through a community-based mass trapping intervention, although further research in different socioecological contexts is needed to clearly define the effectiveness of these strategies. In particular, adulticidal ovitrapping in the form of insecticidal-paint-coated plastic containers was shown to reduce adult *Ae. albopictus* mosquito populations. Larvicide-IGR self-spraying by citizens also showed promising results, although its effectiveness heavily relies on residents’ capacity to identify breeding sites, which is limited [30]. In both cases, low participation rates, misinformation around vector biology, and reduced trap maintenance by participants may have had a major detrimental effect on the conducted interventions. Despite these circumstances, the impact of these strategies on females’ density was relevant, reaching an 83.3% reduction in the case of AOT. Future similar control programs should pay special attention to these aspects before the mobilization of the deployment campaign. Within this context, even though the deployed GAT did not show any effect on mosquito populations, previous research under semi-field [50] and field conditions [43] describes the utility of similar devices as *Aedes* surveillance tools. Additionally, previous reports of effective *Ae. aegypti* reductions through oviposition-based mass trapping in Mexico [51] point to the potential inclusion of these types of tools in control programs. In this sense, these alternatives should be further researched in the future, as being insecticide-free, requiring no electricity, and costing little makes them great candidates for massive distribution in *Aedes*-infested areas.

### 4.7. NESCOTIGER’s Limitations Imply the Need for the Development of Further Similar Researchers in Different Study Areas to Better Define Ae. albopictus Control in Residential Areas

Despite the multifaceted approach and the large number of tools deployed, the study presents several limitations. Notably, the study did not employ ovitraps for eggs collection, which could have provided additional data on population fluctuations and consequently more precision regarding the effectiveness of the intervention. Indeed, ovitraps have proven to be very useful in the Levante region for monitoring mosquito populations [5,52]. Incorporating ovitraps in future studies could enhance the understanding of mosquito population dynamics and improve the overall effectiveness of integrated vector management strategies. Furthermore, the study’s reliance on citizen participation introduced variability in trap deployment and maintenance, potentially affecting the consistency and reliability of the data. In this sense, the dropping out of a citizen in charge of operating one BG-Sentinel in sector 5 implied the need to discard this area from the study. Additionally, due to time limitations, a previous field in-depth inspection of the study area could not be conducted, which led to the elimination of sector 0, as it was later defined during Phase II as a completely different ecological area. In this regard, the effect of SCB treatment could not be properly evaluated, as no negative control was available for its evaluation. Lastly, the study’s geographical scope was limited to a specific residential area, which may not be representative of other regions with different environmental and socio-economic conditions. These limitations suggest the need for more comprehensive and controlled studies to validate these findings.

As such, it is considered that future research evaluating *Ae. albopictus* control in residential areas should focus on the development of an in-depth preliminary field inspection for a better understanding of the terrain, as well as a better characterization of the potential participants, to further increase citizens’ participation rate in any mass trapping intervention.

### 4.8. Social Actors’ Participation Was Essential for the Development of the NESCOTIGER Project

Finally, it should be stated that this research could not have been conducted without the collaboration of helping participants. Within a citizen-science scope [53], the general education of the population around the biology and control of *Ae. albopictus* [30], the deployment and maintenance of control tools, and the special training of a reduced number of participants (n = 6 distribution points and n = 12 monitoring device operators) greatly reduced the project’s personnel costs while enriching the obtained results through the inclusion of the community in the development of this scientific research. Additionally, taking into consideration the recent dramatic events of the “Depresión Aislada en Niveles Altos” (DANA) during the last quarter of 2024 [54], which greatly affected many municipalities, including Torrent, where this research was conducted, and based on the previous reports of high mosquito abundances during 2022 [19], the risk of arboviral transmission [55] needs to be given special attention in residential areas of Valencia.

## 5. Conclusions

This research confirmed the suitability of the control strategy based on coordinated interventions in public and private areas in a community-driven manner supported by an educational campaign. Mass trapping deployment through this approach was proven to be feasible, with the AOT proving particularly effective, as evidenced by the concordance between adult monitoring and reported residents’ perceptions. Nevertheless, further research should confirm the findings of this study in a new large-scale trial following this strategy and using AOTs as the only trapping system evaluated over at least two seasons. Such an extended study would permit proper follow-up regarding mosquito populations and likely lead to increased neighborhood participation.

## Figures and Tables

**Table 1:** General lineal regression model evaluating the effect of the number of deployed mass trapping devices per 150 m radius from BG-Sentinel 2 monitoring devices over the number of captured *Ae. albopictus* females in 48 h. Intercept, estimated captures in the absence of control devices.

Variable	Estimate	Std. Error	*p*
Intercept	11.57	5.09	0.03
GAT	0.15	0.03	<0.001
AOT	−0.09	0.03	<0.01
LAR	−0.03	0.06	0.61
SCB	0.08	0.27	0.77

Adjusted R^2^ = 0.32, *p* < 0.001.

## Data Availability

The data presented in this study are available on request from the corresponding author.

## Appendix A

Perceptions and satisfaction questionnaire
Dear Neighbor:

The NESCOTIGER project is entering its final phase, and data collection on mosquito populations, scupper inspections, and random trap checks is being completed. Evaluation activities will be completed in October, after which the data will be analyzed and conclusions drawn.

The evaluation activities include this survey, which focuses on the residents’ perceptions and satisfaction with various aspects of the project. This activity will take approximately 3–5 min to complete. The specific answers to the questions about the effectiveness of the traps should reflect their overall perceptions over the time the project has been developed, not just at this point in the season.

The questions in this satisfaction survey ask you to rate your satisfaction on a scale of 1 (very poor) to 5 (very good).

Thank you again for your cooperation.

1. Place of residence
  - El Vedat de Torrent
  - Monte Real Urbanization/Tennis Club
  - None of the above
2. Your age
3. Gender
  - Male
  - Female
  - Other
  - Do not know/No answer
4. Citizenship
5. What does the NESCOTIGER project mean to you?
  - A campaign by the Torrent Town Council to reduce the nuisance caused by the tiger mosquito in El Vedat
  - A community activity to eliminate the tiger mosquito problem
  - A scientific study to evaluate the efficacy of traps as a tiger mosquito control tool
  - A tiger mosquito control strategy implemented by a pest control company in El Vedat
  - Do not know/No answer
6. What sector do you belong to and what traps have you been given?
  - Sector 1 = Sticky trap (adhesive tape trap)
  - Sector 2 = Lethal Ovitrap (black container painted on the inside)
  - Sector 3 = Larvicide (spray + black container to be sprayed inside)
  - Sector 4 = Combination of the above traps
  - Sector 5 = No traps
  - Sector 0 = Monte Real and Club de Tenis
  - Do not know/No answer
7. How do you rate the information you have received about the NESCOTIGER project through various channels (website, neighborhood association newsletters, social networks, WhatsApp, town hall, etc.)? (Sectors 1 to 5)
  - 1
  - 2
  - 3
  - 4
  - 5
  - Do not know/No answer
8. How do you rate the attention given to your requests (telephone, WhatsApp messages, email, etc., sectors 1 to 5)?
  - 1
  - 2
  - 3
  - 4
  - 5
  - Do not know/No answer
9. Have you treated the outside of your house with insecticides?
  - Yes, regularly with household insecticides
  - Yes, by a pest control company
  - Yes to both of the above statements (regular use of household insecticides by a pest control company)
  - No.
  - Do not know/No answer
10. Have you taken any measures to reduce the number of breeding sites for tiger mosquitoes (emptying water containers, avoiding the accumulation of water in objects, etc.)?
  - Yes, frequently
  - Yes, regularly
  - No
  - Do not know/No answer
11. Which of the following measures have you taken to reduce tiger mosquito breeding sites in your home?
  - Emptying containers of water regularly
  - Preventing the accumulation of water by protecting or covering elements that could hold water during rainfall
  - Use chemical products on breeding sites
  - Application of hot water to potential breeding sites
  - Other
12. How do you rate the trap delivery process (sectors 1 to 4)?
  - 1
  - 2
  - 3
  - 4
  - 5
  - Do not know/No answer
  - Not applicable (sectors 0 and 5)
13. Have you carried out the maintenance indicated for the siphons (refilling with water, changing the adhesive tape, changing the water, etc.) (sectors 1 to 4)?
  - Yes, sporadically
  - Yes, frequently
  - No
  - Do not know/No answer
  - Not applicable (sectors 0 and 5)
14. Did you keep the traps in operation until the end of the project (October) (sectors 1 to 4)?
  - Yes
  - No
  - Do not know/No answer
  - Not applicable (sectors 0 and 5)
15. How do you rate the effectiveness of the traps in terms of tiger mosquito abundance and perceived bites during the entire project implementation period (April–October) (Sectors 1 to 4)?
  - 1
  - 2
  - 3
  - 4
  - 5
  - Do not know/No answer
  - Not applicable
16. Have you noticed any difference in the abundance of tiger mosquitoes compared to the previous year? (All sectors)
  - Yes, more
  - Yes, less
  - I have not noticed any difference
  - Do not know/No answer
17. Would you use the trap(s) again next year (sectors 1 to 4)?
  - I would
  - Would not
  - Do not know/No answer
  - Not applicable (sectors 0 and 5)
18. Have you noticed any negative effects of the use of traps on your family, pets, or other animals (sectors 1 to 4)? (multiple answers possible)
  - Yes, on humans
  - Yes, on pets
  - Yes, about other animals
  - No
  - Do not know/No answer
  - Not applicable (sector 5)
19. If you have noticed any of the negative effects mentioned above, please describe them, indicating the signs and their intensity:
20. Have you noticed a decrease in the abundance of other pest insects as a result of the use of traps (sectors 1 to 4)? (multiple answers possible)
  - Yes, fewer wasps
  - Yes, fewer flies
  - Yes, fewer other types of insects
  - No
  - Do not know/No answer
  - Not applicable (sectors 0 and 5)
21. What is your overall satisfaction with the NESCOTIGER project (information, communication, effectiveness, effort, etc.)? (Sectors 1 to 4)
  - 1
  - 2
  - 3
  - 4
  - 5
  - Do not know/No answer
  - Not applicable (sectors 0 and 5)

## References

[1] Kraemer M.U., Sinka M.E., Duda K.A., Mylne A.Q., Shearer F.M., Barker C.M., Moore C.G., Carvalho R.G., Coelho G.E., Van Bortel W. (2015). The global distribution of the arbovirus vectors *Aedes aegypti* and *Ae. albopictus*. eLife.

[2] Adhami J., Reiter P. (1998). Introduction and establishment of *Aedes (Stegomyia) albopictus* Skuse (Diptera: Culicidae) in Albania. J. Am. Mosq. Control. Assoc..

[3] European Center for Disease Prevention and Control Aedes Albopictus–Current Known Distribution: July 2024. https://www.ecdc.europa.eu/en/publications-data/aedes-albopictus-current-known-distribution-july-2024.

[4] Aranda C., Eritja R., Roiz D. (2006). First record and establishment of the mosquito *Aedes albopictus* in Spain. Med. Vet. Entomol..

[5] Collantes F., Delacour S., Alarcón-Elbal P.M., Ruiz-Arrondo I., Delgado J.A., Torrell-Sorio J.A., Bengoa M., Eritja R., Miranda M.A., Molina R. (2015). Review of ten-years presence of *Aedes albopictus* in Spain 2004–2014: Known distribution and public health concerns. Parasit. Vectors.

[6] Martínez-Barciela Y., González A.P., Martínez J.M.P., Gradín F.C., González J.G., Costa X.A., Pousa-Ortega A., Íñiguez-Pichele E., Álvarez-Cortiñas M., Eritja R. (2024). Primera cita de *Aedes albopictus* para Galicia, obtenida mediante ciencia ciudadana por Mosquito Alert. Gac. Sanit..

[7] Reiter P. (1998). *Aedes albopictus* and the World Trade in Used Tires, 1988-1995: The Shape of Things to Come?. J. Am. Mosq. Control. Assoc..

[8] Eritja R., Palmer J.R., Roiz D., Sanpera-Calbet I., Bartumeus F. (2017). Direct evidence of adult *Aedes albopictus* dispersal by car. Sci. Rep..

[9] Paupy C., Delatte H., Bagny L., Corbel V., Fontenille D. (2009). *Aedes albopictus*, an arbovirus vector: From the darkness to the light. Microbes Infect..

[10] Gratz N.G. (2004). Critical review of the vector status of *Aedes albopictus*. Med. Vet. Entomol..

[11] Vega-Rúa A., Marconcini M., Madec Y., Manni M., Carraretto D., Gomulski L.M., Gasperi G., Failloux A.B., Malacrida A.R. (2020). Vector competence of *Aedes albopictus* populations for chikungunya virus is shaped by their demographic history. Commun. Biol..

[12] European Centre for Disease Prevention and Control Dengue Outbreak in Madeira, Portugal. https://www.ecdc.europa.eu/sites/default/files/media/en/publications/Publications/dengue-madeira-ECDC-mission-2013.pdf.

[13] Seixas G., Salgueiro P., Bronzato-Badial A., Gonçalves Y., Reyes-Lugo M., Gordicho V., Ribolla P., Viveiros B., Silva A.C., Pinto J. (2019). Origin and expansion of the mosquito *Aedes aegypti* in Madeira Island (Portugal). Sci. Rep..

[14] Hedrich N., Bekker-Nielsen Dunbar M., Grobusch M.P., Schlagenhauf P. (2025). *Aedes*-borne arboviral human infections in Europe from 2000 to 2023: A systematic review and meta-analysis. Travel Med. Infect. Dis..

[15] European Centre for Disease Prevention and Control (2018). Rapid Risk Assessment: Local Transmission of Dengue Fever in France and Spain, 22 October 2018.

[16] Delatte H., Desvars A., Bouétard A., Bord S., Gimonneau G., Vourc’h G., Fontenille D. (2010). Blood-feeding behavior of *Aedes albopictus*, a vector of Chikungunya on La Réunion. Vector Borne Zoonotic Dis..

[17] Reinbold-Wasson D.D., Reiskind M.H. (2021). Comparative Skip-Oviposition Behavior Among Container Breeding *Aedes* spp. Mosquitoes (Diptera: Culicidae). J. Med. Entomol..

[18] Boubidi S., Roiz D., Rossignol M., Chandre F., Benoit R., Raselli M., Tizon C., Cadiou B., Tounsi R., Lagneau C. (2016). Efficacy of ULV and thermal aerosols of deltamethrin for control of *Aedes albopictus* in Nice, France. Parasit. Vectors.

[19] Alarcón-Elbal P.M., López-de-Felipe M., Gil-Torró I., García-Masiá I., Mateo-Herrero P., Bueno-Marí R. (2024). Where does *Aedes albopictus* (Diptera: Culicidae) really breed in a Mediterranean residential area? Results from a field study in Valencia, Eastern Spain. Bull. Entomol. Res..

[20] Abramides G.C., Roiz D., Guitart R., Quintana S., Guerrero I., Giménez N. (2011). Effectiveness of a multiple intervention strategy for the control of the tiger mosquito (*Aedes albopictus*) in Spain. Trans. R. Soc. Trop. Med. Hyg..

[21] Bartlett-Healy K., Hamilton G., Healy S., Crepeau T., Unlu I., Farajollahi A., Fonseca D., Gaugler R., Clark G.G., Strickman D. (2011). Source reduction behavior as an independent measurement of the impact of a public health education campaign in an integrated vector management program for the Asian tiger mosquito. Int. J. Environ. Res. Public. Health.

[22] Baldacchino F., Caputo B., Chandre F., Drago A., della Torre A., Montarsi F., Rizzoli A. (2015). Control methods against invasive *Aedes* mosquitoes in Europe: A review. Pest Manag. Sci..

[23] Bellini R., Albieri A., Carrieri M., Colonna R., Donati L., Magnani M., Pilani R., Veronesi R., Chiot G., Lanza N. (2009). Efficacy and lasting activity of four IGRs formulations against mosquitoes in catch basins of northern Italy. Europ. Mosq. Bull..

[24] Anderson J.F., Ferrandino F.J., Dingman D.W., Main A.J., Andreadis T.G., Becnel J.J. (2011). Control of mosquitoes in catch basins in Connecticut with *Bacillus thuringiensis israelensis, Bacillus sphaericus* and spinosad. J. Am. Mosq. Control. Assoc..

[25] Baldacchino F., Bussola F., Arnoldi D., Marcantonio M., Montarsi F., Capelli G., Rosà R., Rizzoli A. (2017). An integrated pest control strategy against the Asian tiger mosquito in northern Italy: A case study. Pest Manag. Sci..

[26] Perich M.J., Kardec A., Braga I.A., Portal I.F., Burge R., Zeichner B.C., Brogdon W.A., Wirtz R.A. (2003). Field evaluation of a lethal ovitrap against dengue vectors in Brazil. Med. Vet. Entomol..

[27] Shahar M.K., Ismail S., Ahmad R., Omar T. (2022). The effectiveness of MyMAT *Aedes* mosquito trap in reducing dengue cases. J. Vector Borne Dis..

[28] Universitat de Valencia. 2016. Detección de Mosquito Tigre (*Aedes albopictus*) en la Comunitat Valenciana. https://www.dival.es/es/sala-prensa/sites/default/files/sala-prensa/mapa%20actualizado%20a%20septiembre%202016_0.pdf.

[29] Sede Electronica del Catastro. https://www.sedecatastro.gob.es/.

[30] Alarcón-Elbal P.M., López-de-Felipe M., Gil-Torró I., García-Masiá I., Mateo-Herrero P., Bueno-Marí R. (2024). Knowledge, attitude, and practices of adults and children towards the Asian tiger mosquito, *Aedes albopictus* (Diptera: Culicidae), in a recently invaded municipality of Valencia, Spain. Int. J. Trop. Insect Sci..

[31] Hoel D.F., Obenauer P.J., Clark M., Smith R., Hughes T.H., Larson R.T., Diclaro J.W., Allan S.A. (2011). Efficacy of Ovitrap Colors and Patterns for Attracting *Aedes albopictus* at Suburban Field Sites in North-Central Florida. J. Am. Mosq. Control Assoc..

[32] (2004). Global Strategic Framework for Integrated Vector Management.

[33] Pan American Health Organization (2019). Evaluation of Innovative Strategies for Aedes aegypti Control: Challenges for their Introduction and Impact Assessment.

[34] Arana-Guardia R., Baak-Baak C.M., Loroño-Pino M.A., Machain-Williams C., Beaty B.J., Eisen L., García-Rejón J.E. (2014). Stormwater drains and catch basins as sources for production of *Aedes aegypti* and *Culex quinquefasciatus*. Acta Trop..

[35] Pettit W.J., Whelan P.I., McDonnell J., Jacups S.P. (2010). Efficacy of alpha-cypermethrin and lambda-cyhalothrin applications to prevent *Aedes* breeding in tires. J. Am. Mosq. Control Assoc..

[36] Roiz D., Pontifes P.A., Jourdain F., Diagne C., Leroy B., Vaissière A.C., Tolsá-García M.J., Salles J.M., Simard F., Courchamp F. (2024). The rising global economic costs of invasive *Aedes* mosquitoes and *Aedes*-borne diseases. Sci Total Environ..

[37] Montalvo T., Higueros A., Valsecchi A., Realp E., Vila C., Ortiz A., Peracho V., Figuerola J. (2022). Effectiveness of the Modification of Sewers to Reduce the Reproduction of *Culex pipiens* and *Aedes albopictus* in Barcelona, Spain. Pathogens.

[38] Gratz N. (1994). What must be done to effectively control *Aedes aegypti*?. Trop. Med..

[39] European Centre for Disease Prevention and Control (2017). Vector control with a focus on *Aedes aegypti* and *Aedes albopictus* mosquitoes). Literature Review and Analysis of Information.

[40] Donati L., Carrieri M., Bellini R. (2020). A Door-To-Door Strategy for *Aedes albopictus* Control in Northern Italy: Efficacy, Cost-Analysis and Public Perception. Vector Biol. J..

[41] Jaffal A., Fite J., Baldet T., Delaunay P., Jourdain F., Mora-Castillo R., Olive M.M., Roiz D. (2023). Current evidences of the efficacy of mosquito mass-trapping interventions to reduce *Aedes aegypti* and *Aedes albopictus* populations and *Aedes*-borne virus trans-mission. PLOS Negl. Trop. Dis..

[42] Barrera R. (2022). New tools for *Aedes* control: Mass trapping. Curr. Opin. Insect Sci..

[43] López-de-Felipe Escudero M., Rodríguez-Sosa M.A., Alarcón-Elbal P.M. (2025). Comparing captures and efficacy of two commercial gravid traps for characterizing *Aedes albopictus* (Diptera: Culicidae) populations in the Mediterranean basin. Int. J. Pest Manag..

[44] Jahir A., Kahamba N.F., Knols T.O., Jackson G., Patty N.F.A., Shivdasani S., Okumu F.O., Knols B.G.J. (2022). Mass Trapping and Larval Source Management for Mosquito Elimination on Small Maldivian Islands. Insects.

[45] Tilak R., Wankhede U., Mukherjee R. (2022). Novel pyriproxyfen based treatment for *Aedes* breeding control through a long-lasting formulation: Laboratory and field trials in Western Maharashtra, India. J. Vector Borne Dis..

[46] Cárdenas R., Cabrera O.L., Carrillo M.A., Pineda A., Ahumada M.L., Yañez Y., Ismail H., Paine M., Rivera T., Kroeger A. (2024). *Aedes aegypti* control in breeding sites through an insecticidal coating with dual effect: Laboratory trials and safety assessment. Med. Vet. Entomol..

[47] Pinal R., Delacour S., Calvete C., Lucientes J. (2022). The efficacy of microencapsulated biocide paints for the control of *Aedes* (*Stegomya*) *albopictus* Skuse, 1894 under laboratory conditions. Lat. Am. Appl. Res..

[48] Egid B.R., Coulibaly M., Dadzie S.K., Kamgang B., McCall P.J., Sedda L., Toe K.H., Wilson A.L. (2022). Review of the ecology and behaviour of *Aedes aegypti* and *Aedes albopictus* in Western Africa and implications for vector control. Curr. Res. Parasitol. Vector Borne Dis..

[49] Ferrero Gómez L., Ribeiro Rocha H.D., Gil Torró I., Pérez I.S., Mendes D.C., Silva K.L.F., Monteiro D.D.S.R., Dos Reis J.P.T., Leal S.V., Soulé L.F.V. (2024). Insecticide paints: A new community strategy for controlling dengue and Zika mosquito vectors in Cabo Verde. Front. Trop. Dis..

[50] Eiras A.E., Costa L.H., Batista-Pereira L.G., Paixão K.S., Batista E.P. (2021). Semi-field assessment of the Gravid *Aedes* Trap (GAT) with the aim of controlling *Aedes* (*Stegomyia*) *aegypti* populations. PLoS ONE.

[51] Aguilar-Durán J.A., Hamer G.L., Reyes-Villanueva F., Fernández-Santos F.A., Uriegas-Camargo S., Rodríguez-Martínez L.M., Estrada-Franco J.G., Rodríguez-Pérez M.A. (2024). Effectiveness of mass trapping interventions using autocidal gravid ovitraps (AGO) for the control of the dengue vector, *Aedes* (*Stegomyia*) *aegypti*, in Northern Mexico. Parasit. Vectors.

[52] Alarcón-Elbal P.M., Delacour Estrella S., Ruiz Arrondo I., Collantes F., Iniesta J.A., Morales-Bueno J., Sánchez-López P.F., Amela C., Sierra-Moros M.J., Molina R. (2014). Updated distribution of *Aedes albopictus* (Diptera: Culicidae) in Spain: New findings in the mainland Spanish Levante, 2013. Mem. Inst. Oswaldo Cruz..

[53] Den Broeder L., Devilee J., Van Oers H., Schuit A.J., Wagemakers A. (2018). Citizen Science for public health. Health Promot. Int..

[54] (2024). World Meteorological Organization (WMO) Devastating Rainfall Hits SPAIN in Yet Another Flood-Related Disaster. https://wmo.int/media/news/devastating-rainfall-hits-spain-yet-another-flood-related-disaster.

[55] Ceccarelli G., Branda F., Giovanetti M., Ciccozzi M., Scarpa F. (2024). The urgent need for arbovirus surveillance and control following a catastrophic event: The case of the DANA flood event in Valencia. New Microbes New Infect..

